# Understanding Lifestyle Dissonance: A Neurobiological Narrative to Strengthen Preventive Health Behavior

**DOI:** 10.1111/ene.70606

**Published:** 2026-04-21

**Authors:** Daniela Berg, Mareike Krause, Eva Schaeffer, Gesine Hermann

**Affiliations:** ^1^ Department of Neurology Christian‐Albrechts‐University/University Hospital Schleswig‐Holstein Kiel Germany

**Keywords:** adherence, noncommunicable disease, prevention

## Abstract

**Background:**

Noncommunicable diseases are the leading cause of death and disability worldwide, although many of their detrimental health effects can be prevented by lifestyle changes. Despite growing public and individual recognition of the importance of prevention, adherence to recommended measures is limited. We refer to this discrepancy between knowledge and action, specifically in relation to preventive lifestyle behavior, as *lifestyle dissonance*.

**Methods:**

This paper presents a narrative synthesis and aims to provide a conceptual framework for communication with patients and the public that includes neurobiological and evolutionary perspectives. It discusses interactions between neural reward systems, executive control mechanisms, and contemporary environmental triggers, including nutrition, exercise, sleep, and stress, as examples of the six pillars of lifestyle medicine.

**Results:**

We propose that *lifestyle dissonance* can serve as a didactic label in communication with patients and the public to explain the mismatch between evolved neural mechanisms and the modern environment. Rather than a sole failure of willpower, non‐adherence to preventive lifestyle measures is partly founded in a conflict within neural circuitry. Patient education regarding the brain's underlying processes and their influence on behavior has the potential to enable individuals to actively redesign their actions, train their reward systems, and thus reinforce healthy behavior.

**Conclusion:**

Integrating a narrative on neurobiological mechanisms into public health and clinical practice strategies should thus be aimed at helping bridge the gap between intention and sustainable preventive behavior. This conceptual framework provides a basis for supporting patients and the public in achieving lifestyle goals and improving the effectiveness of preventive measures.

## 1 Need and Conceptualization of a Narrative

The rapid and profound rise in chronic noncommunicable diseases (NCDs) constitutes a major global challenge, exerting severe impact on individuals, social environments, healthcare systems, and the socio‐economic stability of nations worldwide. By 2050, NCDs are predicted to account for more than 77% of deaths globally. Today, they are already responsible for the majority of disability‐adjusted life years [1, 2]. According to the WHO, premature deaths due to NCDs, in particular cardiovascular diseases, strokes, and type 2 diabetes, could be significantly reduced by effective primary prevention [3, 4].

As NCD examples from the neurological field, there has been a devastating increase in neurodegenerative diseases. The number of people living with Alzheimer's disease and other forms of dementia is expected to nearly triple worldwide from around 57 million in 2019 to 152 million in 2050 [5]. Importantly, 45% of dementia cases could be avoided if modifiable risk factors were eliminated [5, 6]. Similarly, Parkinson's disease (PD), currently the fastest‐growing neurological disorder globally, is expected to rise from around 11.8 million cases in 2021 to about 25.2 million by 2050, representing a 112% increase [7]. A series of case–control, observational, and consecutive meta‐analytic studies have demonstrated that regular physical exercise significantly reduces the risk of developing Parkinson's disease [7, 8].

Despite growing awareness of the importance and effectiveness of prevention, and despite numerous initiatives, including large‐scale programs [9], the implementation of effective preventive measures on the basis of lifestyle changes remains limited and inconsistent. Many patients and healthy individuals report that they are aware of the importance of a healthy diet, regular physical activity, and sufficient sleep—three major lifestyle fields with preventive potential for most NCDs. However, incorporating these behaviors into daily routines remains challenging for many, and repeated difficulties in implementation entail frustration and reduced self‐efficacy [9].

This persistent gap between knowledge and action [1] aligns with the neuropsychological construct commonly referred to as intention–behavior gap [10]. We conceptualize this gap—specifically in relation to lifestyle behaviors—and the cognitive dissonance it engenders as *lifestyle dissonance*, embedding it within both neuroanatomical and evolutionary frameworks. We propose it as a didactic term that primarily refers to four of the six pillars of lifestyle medicine [10], which build the base of preventive medicine and a healthy life.

In this article, we will focus on the pillars that are most directly modifiable at the individual level: nutrition, exercise, sleep, and stress. These are considered in the light of underlying neurobiological regulatory mechanisms. The pillars of social connection and avoidance of risky substances are not covered here, as social connection relates less directly to neuroanatomical and evolutionary mismatch mechanisms compared to the first four pillars, and avoidance of risky substances introduces additional dimensions of behavioral and physiological dependence that extend beyond the present conceptual focus.

## 2 Approach and Scope

This article provides a narrative synthesis of the neurobiological and evolutionary foundations for addressing *lifestyle dissonance*. We conducted a non‐systematic literature search in PubMed, focusing on the interactions between neuroanatomy (particularly the reward system vs. executive control), evolutionary biology, and behavioral health.

Without claiming to be a comprehensive systematic review, this framework aims to provide a helpful narrative and didactic tool for clinicians and public health experts to support patient and public education and health literacy. To this end, we focus on the most common noncommunicable diseases with high potential for prevention through lifestyle interventions.

It is beyond doubt that lifestyle behaviors are closely linked to socio‐economic contexts, including structural inequalities, poverty, and security issues, which must be addressed as a matter of public policy. Effective implementation of preventive measures, therefore, requires the engagement of numerous stakeholders, including healthcare systems, policymakers, the economy, educational institutions, and the media. At the same time, it is crucial to address the individual, who ultimately must implement preventive measures that require substantial lifestyle changes. In this context, health literacy has emerged as a critical determinant of effective prevention, as strongly supported by the WHO [1, 11]. The framework presented here focuses on factors that patients can influence themselves, providing a biological rationale that reduces stigmatization and empowers individuals to take actionable steps toward healthier behaviors.

From a neurological and neuroscientific perspective, a narrative that helps individuals to understand “why we often don't do what we want to do” may support efforts to maintain a healthy lifestyle in addition to existing knowledge and, more generally, contribute to the development of targeted prevention strategies. The following perspective aims to lead to the comprehension that non‐adherence to preventive measures should not be understood as a failure of willpower or knowledge, but as the result of interactions between evolutionary and individually evolved neural reward systems, executive control mechanisms, and environmental factors exploiting these biological vulnerabilities [9].

## 3 Evolutionary Origins of Lifestyle Dissonance

**FIGURE 1:**
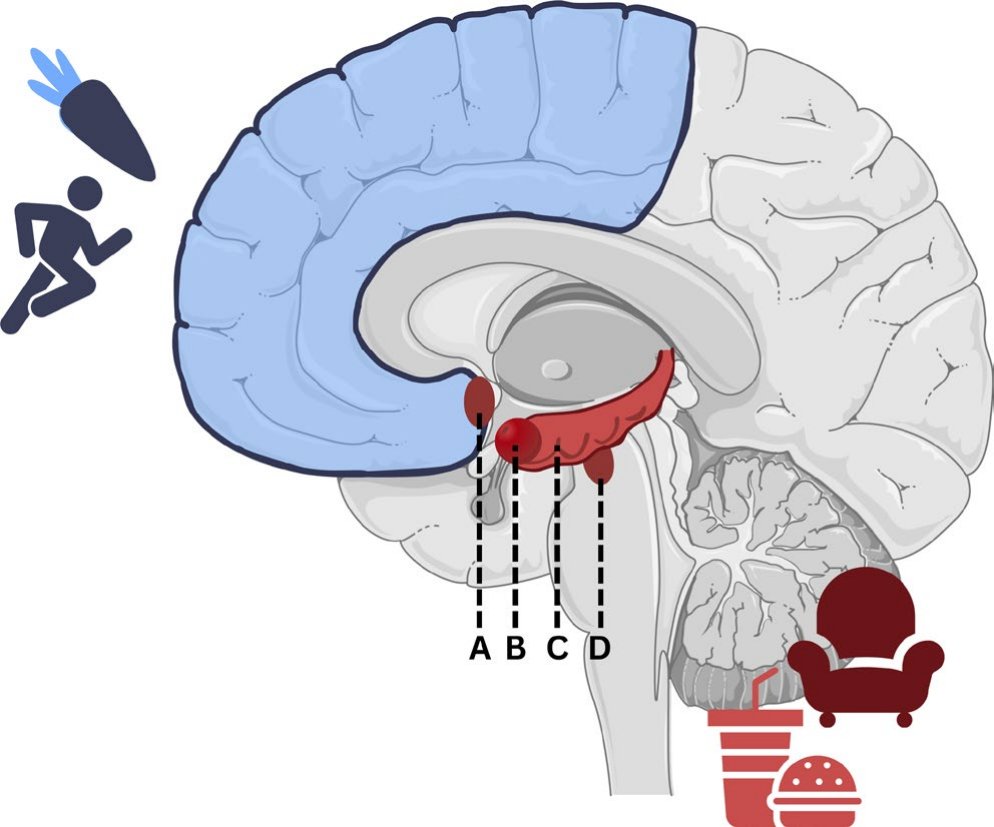
Schematic representation of the brain, focusing on the mesolimbic system (red) and the prefrontal cortex (blue) as the structures relevant to this narrative. The mesolimbic system comprises the nucleus accumbens (A), the amygdala (B), the hippocampus (C), and the ventral tegmental area (VTA, D). This system is evolutionarily older and rewards energy‐saving activities and energy‐rich food intake by releasing dopamine. The prefrontal cortex, as its counterpart, can integrate higher goals into the decision‐making process.

Although neuroscientific inquiry necessarily emphasizes the complexity of the neural circuits underlying responses to environmental stimuli, a patient‐oriented narrative aimed at supporting self‐empowerment must balance precision with accessibility. For this purpose, we focus on the interplay between two key systems: the mesolimbic system (MLS) and the prefrontal cortex (PFC) (Figure 1). This representation captures the core dynamics of a much more complex network, which also involves regions such as the anterior insula, dorsal anterior cingulate cortex, inferior frontal gyrus, ventral striatum, and orbitofrontal cortex, all of which contribute to self‐regulation and inhibitory control [12]. By emphasizing the MLS–PFC interaction, the model provides a comprehensible neurobiological framework for understanding lifestyle dissonance while maintaining fidelity to the underlying complexity.

Evolutionarily, the MLS reacts with direct reward to various, also potentially harmful stimuli, that once signaled survival [13]. In contrast, the PFC performs higher‐level executive control, including aspects of long‐term evaluation and, accordingly, behavioral modification [14]. The MLS is inherently more dominant because of the evolutionarily older circuits, which can only be overcome by applying cognitive effort. The PFC therefore has to specifically combat a variety of evolutionarily grounded temptations, such as high‐calorie food or natural inertia with a tendency toward sedentary activities [13, 14]. These systems exhibit intricate and highly dynamic interactions. In contemporary environments, however, the MLS frequently exerts a functional predominance, reflecting its evolutionary role in resource scarcity, requiring the PFC to exert considerable cognitive effort to overcome these evolutionarily conserved, automatic impulses [13, 14]. The human brain developed under resource scarcity and physical deprivation, favoring neuronal systems that rewarded calorie intake and energy conservation. Food high in sugar and fat signaled survival because of high energy density. As human cultures emerged, particularly in recent decades, a growing part of the world experiences food abundance, whereas evolutionary adaptive reward systems continue to influence behavior [15].

Like the intake of energy‐dense food, physical rest was rare in evolutionary terms, which resulted in the avoidance of unnecessary physical exertion to preserve vital energy reserves. For early humans, the predominant mode of movement was walking, whereas they only resorted to running in response to external stimuli. Even today, the majority of individuals only find it rewarding to start physical activity when tied to achieving another goal or other gratifications. In the absence of these, it often requires the effortful involvement of the PFC to overcome one's own inertia—which only decreases after repeated exercise. In contrast to the evolutionarily difficult start of physical activity, once started, moderate continuation is associated with reward signals that result in a “runner's high” [16, 17, 18]. This process starts after 20–30 min of training and is more pronounced in regularly active people. It is assumed that this delayed reward evolved to motivate endurance hunting and long‐distance running. However, the underlying mechanism is neurobiologically weaker and, above all, much slower than the evolutionary strategy of sedentary behavior [16, 18, 19].

## 4 Challenges of Modern Lifestyle on Sleep Regulation

In our modern societies, we now recognize the synergistic detrimental effects of imbalanced diets and physical inactivity on health. These are compounded by sleep deprivation, as unlike in the past, it is not sunset that determines bedtime but rather many seemingly rewarding activities distracting us from the need to rest and sleep [20, 21].

Although many people state that they should ideally sleep seven to 8 h a night, this rarely corresponds to reality because of occupational demands, social commitments, and personal habits. Increasing use of digital technologies with blue light emissions and continuous cognitive stimulation delays the circadian rise in melatonin and promotes artificial alertness just before bedtime [22], thereby impeding sleep initiation. In addition, bedtime procrastination, that is, deliberately delaying bedtime without any external necessity, has been identified as a failure of self‐control mechanisms in favor of the easy reward of staying up longer [23]. It is motivated by leisure time or the feeling of regained free time after a busy day. Bedtime procrastination involves activities with immediate reward effects, which is balanced out by trivializing chronic sleep deprivation (e.g., that a cup of coffee the next morning will make up for the tiredness) to the disadvantage of a healthy sleep rhythm [23]. Postponing bedtime itself occurs more frequently in individuals suffering from high daily stress and low self‐control, suggesting that what appears to be a simple preference may reflect an exhausted capacity for regulation in contexts of chronic demands [24]. Persistent sleep deprivation impairs executive control, which in turn leads to feedback loops [25].

Focusing on the MLS and PFC and taking into consideration human evolution and the strife for survival, we can come to an understanding of “why we often don't do what we acknowledge to be good for our health”. We emphasize that this neurobiological framework is intended as a didactic tool in the education of patients and the public to reduce self‐blame and promote patient participation. In the following, an explanation of neurobiological mechanisms is presented from a neurological perspective describing the main circuits involved in stimuli reaction, reduced to essentials for a narrative. Examples of unhealthy dietary patterns and physical inactivity are employed, which can easily be linked to the evolutionary perspective and are widely recognized as being among the most prevalent risk factors for NCDs.

## 5 Neurobiological Mechanisms: Reward, Reinforcement, and Self‐Control

We consider the abovementioned fight between the MLS seeking immediate rewards and the PFC aiming for long‐term goals as the core neurobiological mechanism underlying *lifestyle dissonance*.

**FIGURE 2:**
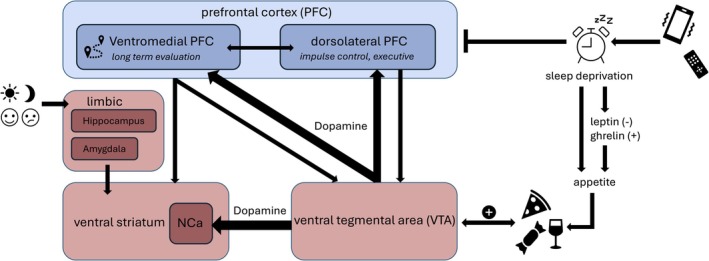
Dynamic interactions among distinct neuroanatomical regions implicated in the formation of habits. The prefrontal cortex (PFC, blue) can be subdivided into a ventromedial and dorsolateral region, both of which interact with the mesolimbic system (MLS, red). The MLS comprises the ventral tegmental area (VTA) and the nucleus accumbens (NCa) as part of the ventral striatum, together with limbic structures such as the hippocampus and amygdala. Consumption of highly palatable foods (e.g., rich in sugar or fat) induces dopaminergic signaling from the VTA to the NCa and PFC. In parallel, excessive media use—via blue light exposure—can disrupt sleep quality, resulting in reduced PFC activity and a relative dominance of MLS function.

Activation of the mesolimbic pathways leads to a release of dopamine, which, by binding to dopamine receptors in the ventral striatum, especially in the nucleus accumbens (NAc), generates a positive feeling of reward, leading to reinforcement of this behavior [13, 26] (Figure 2). (For a better understanding of the neuroanatomical regions implicated in lifestyle dissonance and their functional roles within intention–behavior circuits, please see Table 1.) This activation takes place within a short latency of approximately 200 ms [27]. The immediate benefits of unhealthy behaviors, neurochemically mediated by dopamine release and activation of the GABAergic system, outweigh the more abstract and delayed benefits of healthier ones [13, 26]. Over time, repeated exposure leads to neuroadaptation with sensitization of neuronal circuits for rewards by conditioning the brain to corresponding stimuli. The strength of this neuroadaptation is already shaped by experiences originating in early childhood, such as the use of sweets as sources of comfort or reward. After conditioning, dopamine is released directly when cues associated with the rewarding stimulus occur, for example, the mere smell or sight of a sweet (cue‐induced dopamine release). This leads to an increased craving for the respective consumable and heightened awareness of conditioned stimuli. Beyond dopamine, there are other neuromodulators (e.g., endocannabinoids, opioids, and orexin) interacting with the mesolimbic activity, that in turn contribute to hedonic consumption. They also interact with dopamine signaling directly and further reinforce maladaptive behavior [15, 26].

**TABLE 1:** Neuroanatomical regions implicated in lifestyle dissonance and their functional roles within intention–behavior circuits.

Brain region	Function in decision‐making/reward processes	Example
Amygdala	Processes emotional salience of stimuli, especially fear and reward anticipation	Feeling excitement when anticipating a dessert
Dorsolateral prefrontal cortex (dlPFC)	Supports cognitive control, planning, and suppression of impulsive responses	Resisting the temptation to eat junk food in order to stick to a healthy diet plan
Hippocampus	Contributes to memory‐based decision‐making; links past experiences with current choices	Remembering previous success with exercising regularly and choosing to go to the gym again
Ventral striatum (including nucleus accumbens (NCa))	Encodes reward prediction, motivation, and reinforcement learning; central to dopamine signaling	Dopamine release when anticipating a rewarding activity, strengthening reward‐related learning
Ventral tegmental area (VTA)	Initiates reward prediction and reinforcement learning; dopaminergic projection to the NCa and PFC	Feeling a strong urge to check your phone when you hear a notification sound, because past notifications were rewarding
Ventromedial prefrontal cortex (vmPFC)	Evaluates the subjective value of options; integrates reward and cost information	Weighing the immediate pleasure of eating high‐calorie food against long‐term health goals before deciding what to eat

*Note:* The table summarizes key brain regions involved in lifestyle dissonance, a form of the intention‐behavior gap specific to preventive lifestyle behaviors.

Therefore, dopamine release within the MLS plays a key role in hedonics and operant conditioning. For example, high‐calorie foods or consuming media late at night lead to a strong and rapid activation and thus to the induction of positive emotions [26] (Figure 2). This effect in connection with food intake is particularly evident in obese individuals [28]. They often show increased mesolimbic responsiveness and greater structural connectivity in the VTA‐NAc pathway (VTA—ventral tegmental area, part of the mesolimbic pathway, see Figure 2). Particularly, individuals with a high BMI have increased incentive motivation and neural adaptation as a response to food stimuli [29]. This and the cue‐induced dopamine release can result in consuming without a physiological need and in the context of food in compulsive overeating, a key mechanism in both food and drug addiction [13].

In these scenarios, the PFC, in particular the dorsolateral part (dlPFC), acts as a counterpart of the MLS (Figure 2). It evaluates executive functions, resulting in impulse control, planning for the future, and maintaining goals [14]. Specific cerebral valuation centers, such as the ventromedial PFC (vmPFC), further modulate the way of proceeding so that decisions are taken on the basis of the integration of long‐term goals under consideration of short‐term temptations. Thus, mutual influences and following modulations between dlPFC and vmPFC enable the incorporation of more abstract goals, like “health”, into value‐based decisions [14]. Regarding reaction time, the dlPFC is engaged in early attentional filtering of sensory input (within 150–200 ms of stimulus presentation) to modulate the individual's attention toward goal‐relevant attributes [30]. Afterward (450–650 ms post‐stimulus), integration of multiple signals through later value modulation takes place, influencing the final choice through higher order goals evaluation. Within these signal processing steps, individual differences in activity and connectivity of the dlPFC can be seen [30].

Involvement of the PFC can be visualized by functional imaging. Problems in self‐control occur when different factors—for example, health as a long‐term goal and taste of food as a short‐term goal—must be integrated in the vmPFC for value computation. Here, activation of the dlPFC is associated with modulating decisions in favor of higher‐order long‐term considerations [31]. Individuals with good self‐control show simultaneous vmPFC and dlPFC activation while considering both taste and health attributes in food decision paradigms, whereas poorly self‐controlled individuals that primarily considered taste exhibit vmPFC activity only [31]. The anterior and medial PFC (aPFC and mPFC) further contribute to future‐oriented thinking, playing a role in anticipating delayed rewards, which seems to be more pronounced in self‐controlled individuals [32]. A strong connection between dlPFC and mPFC is positively correlated with good self‐control. That way, long‐term benefits can be prioritized over immediate gratification [33].

## 6 Physiological Factors Influencing Neuronal Control Mechanisms

To engage empathetically with individuals who struggle to implement health‐promoting choices, it is important to address the interaction of lifestyle as discussed above (diet, exercise and sleep) with physiological and additional external factors like stress that hinder decisions the dlPFC would recommend to prefer (Figure 2) [21, 25, 34].

The fourth pillar of lifestyle medicine, that is, stress, is particularly influenced and modulated by physiological mechanisms. Acute and chronic stress compromise the dlPFC's function and its frontostriatal connectivity, impairing working memory and executive functions. This is enhanced by elevated cortisol levels that increase activity in the dorsal striatum. They also reduce connectivity between the hippocampus and amygdala, thereby promoting a shift from cognitive/hippocampal to habitual/striatal control [34, 35]. Acute stress impairs model‐based decision‐making, particularly in people with already chronically elevated stress levels, suggesting that these individuals have depleted the regulatory resources necessary to maintain cognitive control under acute stress [36].

Regarding nutrition and exercise, high sugar consumption can, for instance, lead to a decline in energy level, resulting in lethargy that diminishes the motivation to exercise [37]. Insulin spikes following energy‐rich meals may further increase hunger and thus create a kind of vicious circle [38]. The carbohydrate‐insulin model posits that consumption of high glycemic‐index foods induces postprandial hyperinsulinemia, promoting rapid glucose uptake. This results in a drop of energy and instead triggers increased feelings of hunger [38]. In contrast, a balanced diet stabilizes cerebral glucose levels, thereby supporting sustained prefrontal control and reward regulation.

Regarding sports, homeostatic signals from the body must be overcome (increased heart rate, rapid breathing, and muscle tension), which the brain interprets as stress [39]. By nature, this is considered physically unpleasant. Because short‐term costs (effort, discomfort) are more tangible than the long‐term, abstract benefits (health, mood), motivation often declines in the absence of reinforcing elements [14, 16, 39]. However, as will be discussed below, repetitive training can reset the reward pathways and improve dopaminergic signaling, making it easier to internalize movement as a rewarding behavior.

Similarly, sleep deprivation impairs the activity of the dlPFC and vmPFC (Figure 2), making targeted learning more difficult and increasing the occurrence of habitual behavior [25]. Beyond impairing cognitive control, sleep deprivation has direct metabolic consequences, such as appetite hormone dysregulation, reinforcing unhealthy behaviors by reducing leptin levels (satiety signal) and increasing ghrelin secretion (hunger stimulation). As a consequence, chronic sleep deprivation leads to more hunger and specifically raises the desire for high‐calorie foods [22]. On the other hand, sufficient sleep increases leptin levels and thus suppresses appetite [22]. For the individual, it is important to understand that repeated stress and sleep deprivation enhance lifestyle dissonance in a process called habit formation.

## 7 Habit Formation and Cognitive Control

Sleep deprivation promotes rigid‐habitual responses through activation of the dorsal striatum [34, 35]. Even one night of sleep loss enhances responsiveness to stimuli associated with negative outcomes, suggesting impaired goal‐directed control and increased reliance on habits, likely mediated by reduced vmPFC activation [25].

Habits formed by repetitive behaviors and encoded in the dorsolateral striatum can be executed with minimal cortical input. Energy‐efficient routines are employed in situations of stress and fatigue to conserve cognitive resources for the stressful tasks and staying awake. Thus, strenuous assessment of changing the habit, such as seeking relief of stress or sufficient sleep by the PFC, no longer takes place [25]. This mechanism explains why chronic stress and sleep deprivation often reinforce automatic, maladaptive routines, such as unhealthy eating or physical inactivity. Importantly, stress does not uniformly induce a shift toward habitual responding across individuals. This effect is partially moderated by cortisol reactivity, with pronounced habitual responses observed in cortisol responders [40, 41]. The impact of stress is further modulated by individual differences in basic working memory capacity and stress reactivity according to individual susceptibility, which can then favor impulsive and habitual decision‐making patterns [25, 35]. Chronic stress depletes a person's regulatory resources, which are necessary for maintaining cognitive control under acute stress [36].

Beyond encoded rigid‐habitual responses, their link to environmental influences (e.g., time of day, location, and emotions) and connected routines, mediated by the basal ganglia, must also be taken into account [25, 33]. This process is known as associative learning. In neurobiological terms, rigid patterns, connected to environmental influences, are called “engrammed behaviors”, meaning fixed neuronal representations of a habit, that are able to bypass declarative knowledge [42, 43]. An engram is created when a subgroup of neurons is activated during learning and undergoes lasting chemical and physical changes. These include synaptic plasticity and altered intrinsic excitability. Engrams can be reactivated, for example, to retrieve memories [44, 45]. This can, for example, lead to an automatic search for an (unhealthy) late‐night snack, even if possible negative health consequences are known. In this case, the context (e.g., waking up at night) triggers an automated neural routine. Behavior, engrammed in the dorsolateral striatum becomes more resistant to top‐down control by the prefrontal cortex.

## 8 Being Aware of the Complexity of Lifestyle Dissonance

Although this neurologically based narrative is meant to address the individual and thus aims to increase understanding of the underlying evolutionary and neural mechanisms, it is important to keep in mind and acknowledge that many additional factors contribute to *lifestyle dissonance*.

Beyond evolutionary explanations and mechanisms such as conditioned reactivity of the MLS to energy‐dense food, socioeconomic factors also contribute significantly [46, 47]. Lower socioeconomic status is associated with altered striatal reward processing. Children from disadvantaged neighborhoods show reduced reward‐related activation [46, 48], and neighborhood‐level disadvantage independently predicts reduced connectivity between ventral striatum and PFC, even after controlling for household income [46]. Furthermore, lower socioeconomic status is associated with increased food reward value, leading to a preference for high‐calorie foods, independent of current financial resources [49]. MLS responsiveness is therefore also modulated by socioeconomic determinants. As examples that can be extended: dietary choices are constrained by costs, opportunities for physical activity by access, and lifestyle behavior more broadly by demands of work and caregiving [50]. Effective measures must consider both individual understanding and broader environmental changes needed to promote healthy choices. These determinants need to be considered and addressed in clinical practice and health policy. However, a comprehensive discussion lies beyond the primary scope of this narrative, which focuses on self‐empowerment and the behaviors that individuals can actively influence.

## 9 Conclusion and Translation Into a Narrative

Many individuals are able to describe the principles of a healthy lifestyle and, in general, agree that it is important to adhere to it. However, translating this knowledge into action remains challenging for the majority. A neurological and neuroscience perspective can contribute to the understanding that changing lifestyle habits is not merely a matter of willpower, particularly when educating patients about lifestyle measures for prevention. Rather, this behavior reflects a complex interplay of deep‐rooted neurobiological and evolutionary mechanisms, enforced by parenting and education routines, that favor immediate rewards over abstract long‐term goals (e.g., good health) [15, 51]. This interaction applies to all habits, although additional factors such as genetic predisposition or physical dependencies must also be taken into account.

We propose the term *lifestyle dissonance* as a didactic label for communication with patients and the broader public and empowerment to grasp the conflict between what we know and what we do. Although this phenomenon overlaps with established concepts (e.g., intention‐behavior gap, cognitive dissonance, and self‐regulation failure), *lifestyle dissonance* is specifically intended to provide patients with a neurobiologically based, non‐stigmatizing explanation that can make it easier for them to implement behavioral changes addressing key aspects of a preventive lifestyle. Especially in times of stress and fatigue, we are more susceptible to our conditioned reward pathways, habit loops, and the limited influence of cognitive control [25, 34, 35].

Recognizing these mechanisms enables a more compassionate and biologically informed approach to behavioral change, benefiting patients, the broader population, and of course ourselves. We hypothesize that teaching these neurobiological principles may improve treatment adherence by reducing self‐blame and increasing self‐efficacy. Further research will be needed to test this. Effective strategies should extend rational knowledge transfer to target underlying brain dynamics. Reward systems should be systematically trained to positively reinforce healthy habits. Furthermore, a supportive environment can encourage good decision‐making and promote self‐regulation techniques [14, 33, 34].

Sustainable behavioral change takes time and resilience when temporary failures occur. Our goal as neurologists and health professionals should be the improvement of adherence by communicating the biological principles outlined above. On the basis of a comprehensive understanding of the neurophysiological mechanisms described, we propose to communicate the following three “golden rules” with patients to support behavioral change. (A more detailed example for practical implementation in everyday clinical practice with supporting figures can be found in the appendix.)

1. Be patient: Forming new habits requires many repetitions. Programmed mechanisms only change slowly, as they have usually been developed and maintained over years. A change is possible, even if it requires considerable patience.
2. Be understanding: We should recognize that we instinctively fall back into old routines when we are tired or stressed, without blaming ourselves for it. A temporary relapse into old routines does not mean that one is unable to change behavior permanently. Instead, work on realistic expectations by applying the understanding of the underlying mechanisms.
3. Plan ahead: In times of rest and relaxation, it is easiest to cognitively control our habits. Therefore, it is helpful to make decisions in advance (e.g., writing shopping lists, scheduling exercise sessions, establishing sleep routines) to overcome vulnerable moments.

Many studies have demonstrated that educational programs aimed at improving health literacy yield promising results, potentially leading to more sustainable health‐related choices and behaviors [52, 53]. Knowledge transfer is therefore a central pillar of prevention. From a neurological perspective, promoting an understanding and acceptance of neurobiological limitations—while fostering the deliberate use of their advantages—may constitute an important additional factor for preventing or positively influencing the course of avoidable NCDs, including the increasing burden of neurodegenerative disorders like Alzheimer's and Parkinson's disease.

Additional material related to this article can be found in the appendix, which includes illustrations that may be helpful in communication between healthcare providers and patients. We encourage readers to consult this section for further insights.

## Author Contributions

**Mareike Krause:** writing – original draft, writing – review and editing, investigation. **Gesine Hermann:** investigation, writing – review and editing. **Daniela Berg:** conceptualization, investigation, writing – original draft, supervision, writing – review and editing. **Eva Schaeffer:** supervision, writing – review and editing.

## Conflicts of Interest

The authors declare no conflicts of interest.

## Supporting information


**Figure S1:** Activity within the ventral tegmental area (VTA) activates the nucleus accumbens, a key component of the limbic system, through the mesolimbic dopaminergic pathway, while simultaneously modulating the prefrontal cortex via dopaminergic projections. When activity within these circuits becomes biased toward mesolimbic processes, immediate rewards—such as those elicited by the consumption of high‐fat or high‐sugar foods—tend to override the pursuit of long‐term goals.
**Figure S2:**. In untrained individuals, physical exercise initially elicits a stress‐like physiological response, characterized by increases in blood pressure, heart rate, and respiratory rate. Concurrently, this process is substantial energy consuming. From an evolutionary perspective, no intrinsic reward system has developed to reinforce such activity. Consequently, the combination of physiological stress, and the absence of immediate reward signaling by dopaminergic activation in the mesolimbic pathway contributes to the perception of exercise as an unpleasant experience.
**Figure S3:** Sleep affects the mesolimbic and mesocortical dopaminergic systems. Lack of sleep leads to increased activation of the mesolimbic pathway, both directly and indirectly through a decrease in leptin hormone levels and an increase in ghrelin, which also leads to self‐sustaining of the pathway by stimulating appetite. With sufficient and adequate sleep, the balance shifts in favor of prefrontal cortex activity.

## Data Availability

Data sharing not applicable to this article as no datasets were generated or analysed during the current study.
