# Supplementary material for: Understanding Lifestyle Dissonance: A Neurobiological Narrative to Strengthen Preventive Health Behavior
Source: Eur J Neurol. 2026 Apr 21;33(4):e70606. doi: 10.1111/ene.70606 (PMC13098076; doi:10.1111/ene.70606)
Supplement: Supplementary file 1 — Figure S1: Activity within the ventral tegmental area (VTA) activates the nucleus accumbens, a key component of the limbic system, through the mesolimbic dopaminergic pathway, while simultaneously modulating the prefrontal cortex via dopaminergic projections. When activity within these circuits becomes biased toward mesolimbic processes, immediate rewards—such as those elicited by the consumption of high‐fat or high‐sugar foods—tend to override the pursuit of long‐term goals. Figure S2:. In untrained individuals, physical exercise initially elicits a stress‐like physiological response, characterized by increases in blood pressure, heart rate, and respiratory rate. Concurrently, this process is substantial energy consuming. From an evolutionary perspective, no intrinsic reward system has developed to reinforce such activity. Consequently, the combination of physiological stress, and the absence of immediate reward signaling by dopaminergic activation in the mesolimbic pathway contributes to the perception of exercise as an unpleasant experience. Figure S3: Sleep affects the mesolimbic and mesocortical dopaminergic systems. Lack of sleep leads to increased activation of the mesolimbic pathway, both directly and indirectly through a decrease in leptin hormone levels and an increase in ghrelin, which also leads to self‐sustaining of the pathway by stimulating appetite. With sufficient and adequate sleep, the balance shifts in favor of prefrontal cortex activity. [file ENE-33-e70606-s001.docx]

**Appendix**

**Example of a structured, patient-centered way of communicating the narrative to patients.**

*“Changing your habits and behaviors to improve your health and protect yourself from possible future diseases takes time and patience. It is already a big step to get to the point of wanting to change. Don’t be disappointed or frustrated that so far you may not have been able to implement regular sports and a healthy diet into your daily routine. In fact, this is very understandable as our brain has been trained differently during evolution and our own personal history. There are two main systems in our brain that regulate our behavior. The first one is, in evolutionary terms, much older and requires immediate reward while the other one is more future-oriented and strives for higher goals like good health. However, the older system is also a lot faster in sending signals than the other one and therefore can be more dominant if we do not recalibrate our brain to work more in favor of the younger system. This is quite laborious but it can be achieved and it can, in the long run, prevent a lot of diseases that are connected to our lifestyle. Three golden rules that may help you on your way:*

1. *Be patient: Forming new habits requires many repetitions. Programmed mechanisms only change slowly, as they have usually been developed and maintained over years. A change is possible, even if it requires considerable patience.*
2. *Be understanding: We should recognize that we instinctively fall back into old routines when we are tired or stressed without blaming ourselves for it. A temporary relapse into old routines does not mean that one is unable to change behavior permanently. Instead, work on realistic expectations by applying the understanding of the underlying mechanisms.*
3. *Plan ahead: In times of rest and relaxation, it is easiest to cognitively control our habits. Therefore, it is helpful to make decisions in advance (e.g. writing shopping lists, schedule exercise sessions, establish sleep routines) to overcome vulnerable moments.”*

**Examples of graphics to support understanding of the underlying mechanisms of lifestyle dissonance.**

1. **Dietary habits**


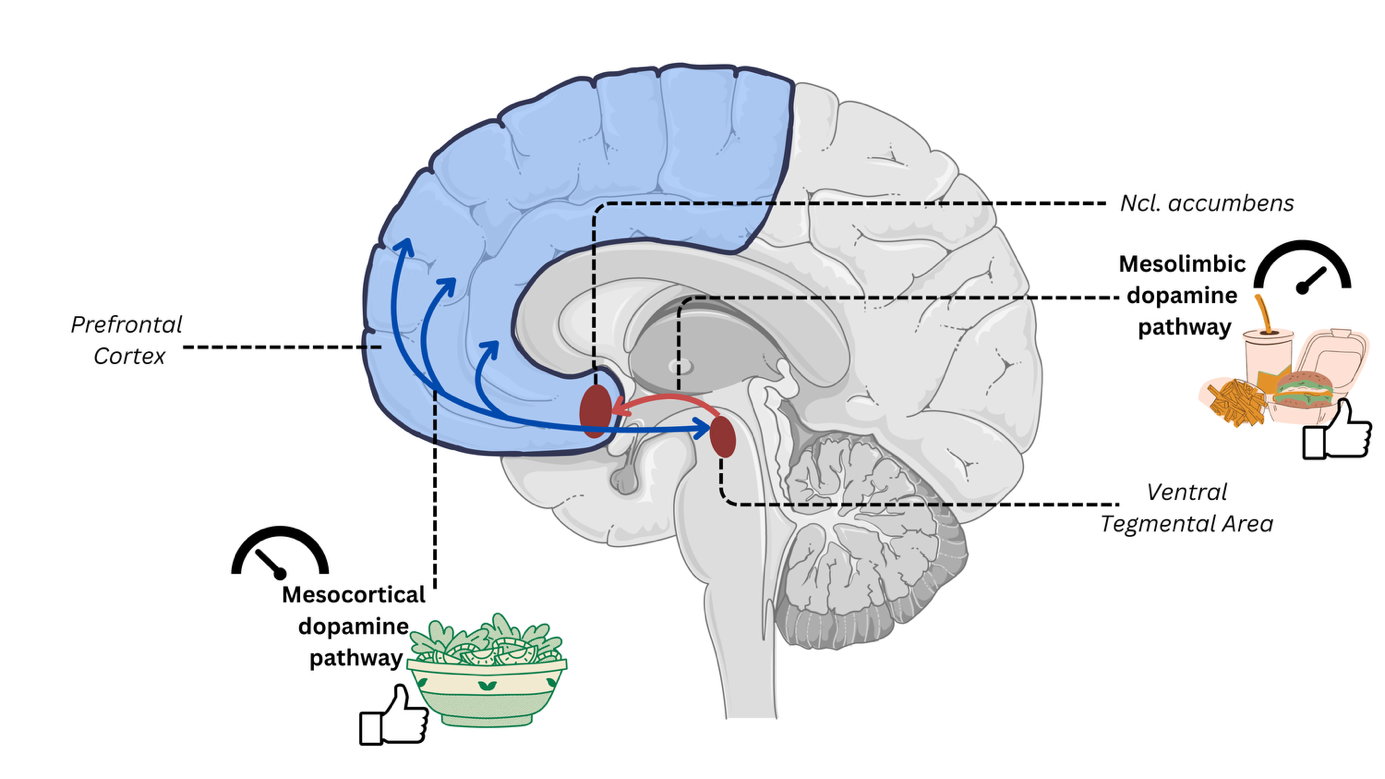


**Supplementary Figure 1**: Activity within the ventral tegmental area (VTA) activates the nucleus accumbens, a key component of the limbic system, through the mesolimbic dopaminergic pathway, while simultaneously modulating the prefrontal cortex via dopaminergic projections. When activity within these circuits becomes biased toward mesolimbic processes, immediate rewards—such as those elicited by the consumption of high-fat or high-sugar foods—tend to override the pursuit of long-term goals.

1. **Physical activity**


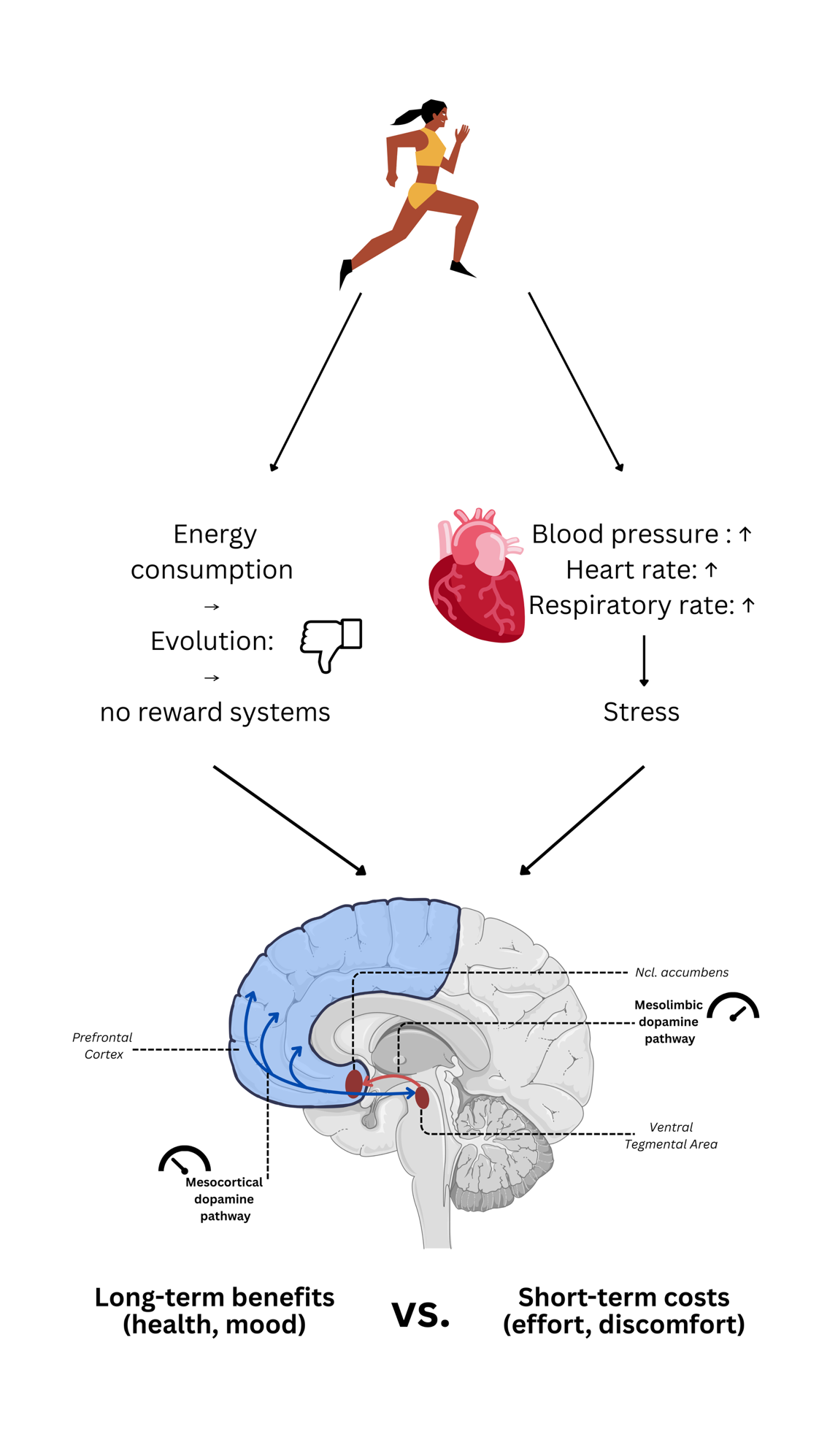


**Supplementary Figure 2**: In untrained individuals, physical exercise initially elicits a stress-like physiological response, characterized by increases in blood pressure, heart rate, and respiratory rate. Concurrently, this process is substantial energy consuming. From an evolutionary perspective, no intrinsic reward system has developed to reinforce such activity. Consequently, the combination of physiological stress, and the absence of immediate reward signaling by dopaminergic activation in the mesolimbic pathway contributes to the perception of exercise as an unpleasant experience.

1. **Sleep**


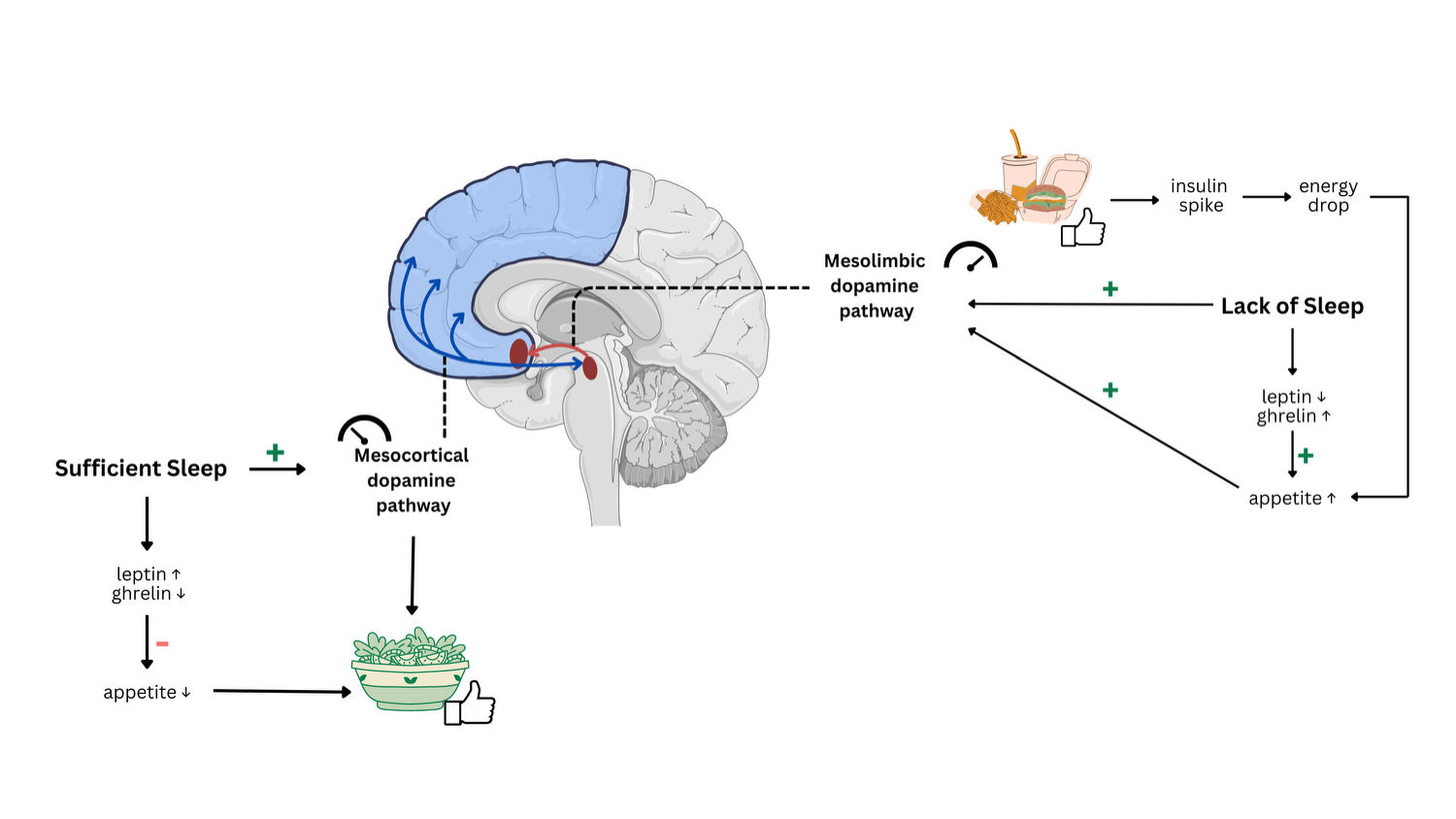


**Supplementary Figure 3**: Sleep affects the mesolimbic and mesocortical dopaminergic systems. Lack of sleep leads to increased activation of the mesolimbic pathway, both directly and indirectly through a decrease in leptin hormone levels and an increase in ghrelin, which also leads to self-sustaining of the pathway by stimulating appetite. With sufficient and adequate sleep, the balance shifts in favor of prefrontal cortex activity.
